# Mapping the functional interactions at the tumor-immune checkpoint interface

**DOI:** 10.1038/s42003-023-04777-3

**Published:** 2023-04-27

**Authors:** Behnaz Bozorgui, Elisabeth K. Kong, Augustin Luna, Anil Korkut

**Affiliations:** 1 Department of Bioinformatics and Computational Biology, UT MD Anderson Cancer Center, Houston, TX 77030 USA; 2 Department of Statistics, Rice University, Houston, TX 77030 USA; 3 Department of Data Sciences, Dana Farber Cancer Institute, Boston, MA 02215 USA; 4 Department of Systems Biology, Harvard Medical School, Boston, US

**Keywords:** Computational biology and bioinformatics, Cancer

## Abstract

The interactions between tumor intrinsic processes and immune checkpoints can mediate immune evasion by cancer cells and responses to immunotherapy. It is, however, challenging to identify functional interactions due to the prohibitively complex molecular landscape of the tumor-immune interfaces. We address this challenge with a statistical analysis framework, immuno-oncology gene interaction maps (ImogiMap). ImogiMap quantifies and statistically validates tumor-immune checkpoint interactions based on their co-associations with immune-associated phenotypes. The outcome is a catalog of tumor-immune checkpoint interaction maps for diverse immune-associated phenotypes. Applications of ImogiMap recapitulate the interaction of SERPINB9 and immune checkpoints with interferon gamma (IFNγ) expression. Our analyses suggest that CD86-CD70 and CD274-CD70 immunoregulatory interactions are significantly associated with IFNγ expression in uterine corpus endometrial carcinoma and basal-like breast cancer, respectively. The open-source ImogiMap software and user-friendly web application will enable future applications of ImogiMap. Such applications may guide the discovery of previously unknown tumor-immune interactions and immunotherapy targets.

## Abstract (web summary)

A statistical analysis framework, termed immuno-oncology gene interaction maps (ImogiMap) provides insights into the tumor immune environment.

## Introduction

Genomically-targeted therapies and immunotherapies have led to improved patient survival in diverse cancer types^1^. Combination therapies that target oncogenic processes and immune evasion may induce more durable responses or even curative effects in select cancer types^2^. It is, however, highly challenging to implement precision therapies involving immunotherapies due to the complexity of the molecular landscape of response predictors.

Identification of biologically relevant tumor-immune interactions that mediate immune evasion by cancer cells may facilitate the discovery of therapeutic targets. For example, the expression of SERPINB9, a potential drug target and a member of the T-cell dysfunction signature genes, is upregulated in tumor cells by interferon gamma (IFNγ, encoded by the *IFNG* gene) in the tumor microenvironment^3,4^. High expression of SERPINB9 confers resistance to CTLA4 checkpoint inhibition and therefore justifies the therapeutic benefit of co-targeting SERPINB9 and CTLA4. In another example of tumor-immune interactions, DNA repair deficiencies can mediate increased vulnerability to immune checkpoint inhibition through the accumulation of mutation loads leading to neoantigens or activation of the STING-pathway^5,6^. Despite such findings, the interactions at the tumor-immune interface, which may inform effective therapies and immune states are relatively unexplored.

The efficacy of immunotherapy agents may depend on diverse molecular and cellular factors including immune infiltration and cell types within the tumor niche, mutational load and tumor foreignness, tumor differentiation states (e.g., epithelial vs. mesenchymal), presence of immune checkpoints, and sensitivity to immune effectors (e.g., IFNγ)^7^. It is time and resource-consuming to search and identify combinatorial tumor-immune interactions through cell biology studies. Infiltration of tumor niches by immune cells usually leads to the enrichment of a large number of coexisting and likely redundant immune checkpoints which obscures the discovery of immune evasion drivers and the selection of immunotherapy targets. Therefore, a naïve analysis of immune checkpoint expression and immune cell identities is partially predictive if not totally futile for the identification of therapeutically actionable drivers of immune evasion^8,9^. It is also critical to identify the tumor-immune interactions that are relevant across large patient cohorts to justify future drug development and clinical testing efforts. At least partly due to the noted challenges, the clinically approved immune checkpoint therapies have remained limited to anti-PD-L1/PD1 and anti-CTLA4 as well as the anti-LAG3, which was recently approved for the treatment of melanoma patients^10^.

Single cell sequencing technologies are useful in decoding the heterogeneity of tumor-immune interactions, yet usually limited in sample volumes (e.g., number of samples, number of cells per sample, depth of sequencing). Due to the small sample volumes, building single cell data-driven models with high statistical and predictive power remains a hard problem^11^. Similarly, emerging technologies for spatially resolved omics profiling are highly promising yet suffer from limited sample volume as well as proteomic and transcriptomic coverage^12,13^. Therefore, computational methods that can infer tumor-immune interactions from bulk RNA expression data is highly desired and could be useful to identify precision immunotherapies tailored to molecular profiles of tumors of cancer patients. Existing tools such as TIMER and CIBERSORT have been very effective in deciphering the immune signatures in tumors as well as exploring interactions between immune features and the expression of individual genes^14,15^. There are, however, no available bioinformatics tools with rigorous statistical validation to investigate the combinatorial tumor-immune interactions that drive immune phenotypes.

Here, we introduce Immuno-oncology gene interaction Maps (ImogiMap), a bioinformatics method, tool, and web application to automate combinatorial searches for interactions between tumor-associated and immune checkpoint processes. The method generates statistically validated interactions between oncogenic events and immune checkpoints that are co-associated with likely predictors of immunotherapy responses. We have developed the ImogiMap based on the rationale that immune checkpoints that associate with both tumor-related processes and immune phenotypes are more likely to be drivers of immune evasion and therapeutic targets compared to the alternatives without such co-associations. Applications in uterine corpus endometrial carcinoma and basal-like breast cancers (TNBC) nominated tumor-immune interactions, that co-associate with interferon gamma (IFNγ), the key cytokine and effector of antitumor immunity^16^. The R-package (https://github.com/korkutlab/imogimap) and the web interface (https://bioinformatics.mdanderson.org/apps/imogimap/) may enable rapid adoption of the technology by translational and basic researchers. The method may guide the identification of critical immune checkpoints and inform therapeutic targets within tumors that manifest the tumor-immune interactions.

## Results

### Immuno-oncology gene interaction maps (ImogiMap)

**Fig. 1:**
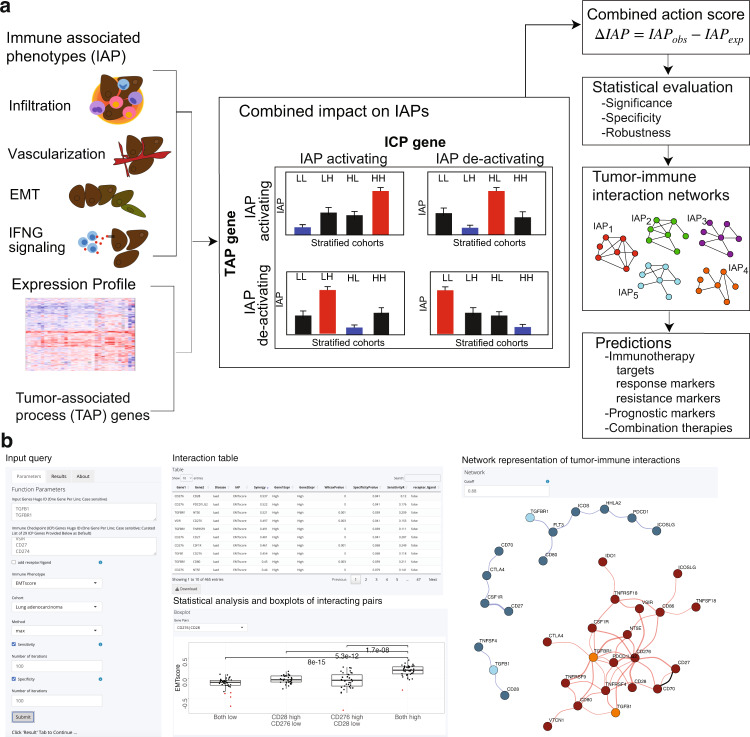
Overview of Imogimap. **a** Immune-associated phenotype (IAP) levels and mRNA expression values are the inputs to ImogiMap. Based on the variation in IAP levels across patients, the algorithm calculates combined action scores between tumor-associated processes (TAP) that may constitute a functional gene signature and therapeutically actionable immune checkpoint (ICP) genes. For each TAP-ICP gene pair, the patient cohort is stratified into four sub-cohorts (LL, LH, HL, HH), and IAP levels are measured within each sub-cohort. A baseline sub-cohort (marked blue) and a target group (marked red) are determined based on four null assumptions on the relationship of the two genes with the IAP (both activating, both deactivating, and one activating while the other deactivating) (see “Methods”). A combined action score under each assumption is calculated and their maximum is reported as the combinatorial association of the TAP-ICP pair with the IAP. For each IAP, a network of gene interactions from specific, robust, and significant interactions is constructed to reflect combinatorial relations between TAPs and ICPs in the context of an IAP. **b** ImogiMap web interface enables queries for oncogenes, immune checkpoints, and immune phenotypes for each 33 cancer types. The outputs are an immune-tumor interactions table with statistical validations (significance, specificity, robustness), network models of tumor-immune checkpoint interactions, and statistical significance analysis across patient cohorts for each interacting pair.

ImogiMap enables statistical and network analysis of combinatorial interactions between Immune checkpoint (ICP) genes, tumor-associated process (TAP) genes, and immune-associated phenotypes (IAPs) (Fig. 1a). TAP genes may be any set of user-defined genes that are expressed in tumor ecosystems (likely but not necessarily within the tumor cells) and with functions involving hallmarks of cancer (e.g., proliferation, apoptosis, DNA repair, tumor metabolism, immune evasion)^17^. TAP genes include but are not necessarily limited to previously defined oncogenes and tumor suppressor genes. ICPs are therapeutically actionable immune checkpoints, for which targeting strategies are in clinical use or trials^18^. The ICP list is provided within ImogiMap and can be updated by the users (Supplementary Table 1). We define IAPs as any quantifiable immune-associated event that can potentially modulate tumor immunity and responses to immunotherapy. In our framework, IAP levels serve as a metric to infer the potentially functional ICP-TAP interactions. Here, we have focused on enrichment of immune cell types that may have differential impact on immune regulation as well as immune cell infiltration (leukocyte fraction)^19^, tumor mutation burden^20^, epithelial-mesenchymal transition (EMT) status^21^, vascularization^22^, T-cell inflammation signature^23^, and IFNγ expression^16^ (Supplementary Tables 2 and 3). Indeed, the choice of IAP depends on disease cohort, therapy type, and the oncogenic processes. ImogiMap is flexible and end users can use the platform to incorporate any quantifiable IAP with evidence of immune-tumor interactions. The outcome of the analysis is connections between TAPs and ICPs that guide selection of possible modulators of immune phenotypes, prognostic markers, and drug targets in the context of therapy response predictors (i.e., IAPs).

The minimally required input to ImogiMap is the mRNA expression data for ICP and TAP genes, and data (e.g., RNA-seq, histological) that quantify IAPs from matched samples. ImogiMap quantifies the tumor-immune interactions with a combined action score between TAP and ICP genes based on their co-association with the IAP of interest. The combined action scores are reminiscent of the synergy scores that are commonly used to define drug-drug interactions based on phenotypic impact of drugs^24^. To calculate the combined action score, the patient cohort is stratified into four sub-cohorts (low–low, high–low, low–high, high–high) based on TAP and ICP gene co-expression levels. Combined action scores are then quantified based on the IAP levels within the sub-cohorts. Alternatively, the mutation status can be entered to define stratifications (e.g., T53-mutated vs. TP53 wildtype). A high combined action score, which is a non-additive deviation of the IAP levels within any one of the sub-cohorts with respect to other sub-cohorts, indicates an interaction between the TAP and the ICP (see “Methods” and Fig. 1). Combined action scores can be calculated using either a highest single agent (HSA) effect^24^ or a more stringent independence model^25^ (Eqs. (5) and (6), both of which are available as options within the ImogiMap R-package and web interface (Fig. 1b). A phenotype may be associated with overexpression or loss of expression (complete or partial) of a TAP or ICP gene. As a result, the non-additive deviations in IAP levels may be observed in any of the four stratified sub-cohorts enabling flexible analyses and interpretations (see Fig. 1 and “Methods”).

### Statistical evaluation and network models of tumor-immune interactions

To filter out potential false predictions, each interaction is statistically evaluated for robustness, statistical significance, and specificity. The robustness metric evaluates the stability of the interactions against moderate changes in the exact data configuration. To assess the robustness, we compute the combined action scores using datasets of partial cohorts (default: 70% coverage, *N*_sampling_ = 1000) that are randomly sampled from the complete cohort. The interaction robustness is quantified as the normalized root mean square deviation of the scores computed from the complete patient cohort vs. partial cohorts. The statistical significance is determined with a Wilcoxon signed-rank test that compares the IAP measurements across the patient sub-cohorts. The null hypothesis for significance is that the median IAP in a “target” sub-cohort and at least one of the remaining three sub-cohorts are sampled from an identical population (see Fig. 1 and “Methods” for details). The interaction specificity is quantified by calculating the *p* values of the combined action scores against a null distribution of scores for non-specific interactions. The null hypothesis for specificity evaluation is that the combined action score between a pair of TAP and ICP genes is equal to the mean scores of either TAP or ICP against a set of randomly selected genes. The null distribution of non-specific interactions is built as combined action scores between each of the genes of interest (TAP and ICP) and randomly sampled genes (*N* = 1000) for a particular IAP. When all statistical validation steps are utilized, the resulting *p* values for significance and specificity can be corrected for multiple hypothesis testing using the Benjamini-Hochberg method. A robust combined action score (not sensitive to moderate changes in data) with high significance (low *p* value from a Wilcoxon signed-rank test) and high specificity (low *p* value against a null model of non-specific interactions) indicates a potentially functional interaction between tumor and immune genes for the IAP of interest.

Through integration of the significant, specific, and robust interactions, a collection of network models is constructed to map the associations of tumor-associated and immune checkpoints in the context of diverse immune phenotypes (Supplementary Fig. 1). We also incorporate the receptor-ligand interaction information based on the annotations in the CellPhoneDB database^26^. Each network model maps multi-faceted interactions between tumor and immune processes as well as ICP receptor-ligand interactions that associate with a significant change in a particular IAP. The collection of maps for diverse phenotypes presents a comprehensive atlas for comparative analysis among several IAPs. We also compare survival of patients whose tumors differentially express the interacting TAP and ICP genes to provide additional evidence on oncogenic relevance of phenotypic impacts. The resulting atlas informs on immune phenotypes, patient survival, and through interpretation of the maps, selection of potential immunotherapy options within patient cohorts carrying the tumor-immune interactions.

### T-cell dysfunction and immune checkpoint interactions in endometrial carcinoma

To validate our tool in a biologically relevant context, we explored the interactions between T-cell dysfunction signature genes and ICPs at the tumor-immune interface. The T-cell dysfunction signature has been previously reported as a potential biomarker to predict response to immunotherapy in select cancer types including Uterine Corpus Endometrial Carcinoma (UCEC) and breast cancer^3^. Specifically, SERPINB9 which is part of the T-cell dysfunction signature stood up as a driver of immune checkpoint inhibitor resistance and is induced in response to IFNγ secretion. Here, we used 26 core T-cell dysfunction signature genes (Supplementary Tables 4 and 5) as the TAP for evaluations against 29 ICP genes in ImogiMap (Supplementary Table 1) in UCEC. To extend our analysis to receptor-ligand pairs, we used the CellPhoneDB database to include additional 23 genes that correspond to receptors or ligands of the previously defined ICPs and T-cell dysfunction genes (Supplementary Table 6). We focused on the *IFNG* gene expression whose gene product, IFNγ protein, and downstream signaling elements are critical for both antitumor immunity and immunotherapy responses^23,27^. The IFNγ protein has dual anti- and pro-tumor roles as it has cytotoxic effects on tumor cells through IFNγ receptor and yet induces expression of pro-tumor, immune suppressive checkpoints such as PD-L1^28^.

**Fig. 2:**
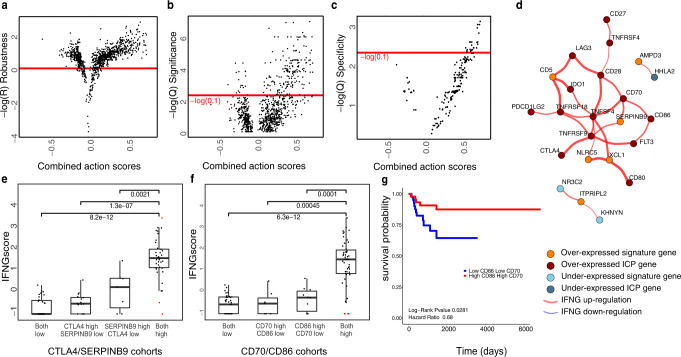
Interactions of T-cell dysfunction signature genes with immune checkpoints in uterine corpus endometrial carcinoma (UCEC). ImogiMap-based assessment of combinatorial interactions for T-cell dysfunction signature genes in UCEC, and therapeutically actionable ICPs, based on their associations with the *IFNG* gene expression. The analysis is based on RNA expression data from tumors of 370 patients. **a** Combined action score robustness. A normalized root mean square deviation (RMSD) is calculated for each combined action score through random sampling (1000x) of sub-cohorts with 70% coverage of the complete cohort. Scores with high robustness, $-{{{{{\rm{log}}}}}}\left({R}\right)\, > \,0$ (red horizontal line) are selected for further analysis (see “Methods”). **b** Statistical significance of combined action scores. An FDR (Benjamini-Hochberg (BH) method) corrected ${Q}$-value based on the Wilcoxon signed-rank test is calculated for robust scores. Scores with ${Q}_{\_{{{{{\rm{Significance}}}}}}}\, < \,0.1$ are selected for further analysis. **c** A BH-corrected *Q*-value for specificity is calculated for each robust and significant interaction (see “Methods”). Scores with ${Q}_{\_{{{{{\rm{Specificity}}}}}}}\, < \,0.1$ are selected for further analysis. **d** The graphical network representing robust, significant, and specific combinatorial associations with IFNγ levels (represented by *IFNG* gene expression). Red (Blue) edges represent upregulation (downregulation) of *IFNG*. Dark red (Dark blue) vertices identify overexpression (low-expression) of ICP genes and orange (blue) vertices identify overexpression (low-expression) of T-cell dysfunction signature genes. **e** Levels of *IFNG* gene expression in TCGA UCEC samples, stratified based on SERPINB9 and CTLA4 levels. *p* values from Wilcoxon signed-rank test indicate the statistical significance of differences in IFNγ levels within the stratified sub-cohorts. **f** Levels of *IFNG* gene expression in TCGA UCEC samples, stratified based on CD86 and CD70 levels. **g** Kaplan–Meier survival curve for UCEC patients stratified by low/high expression of CD70 and CD86.

We identified the significant, specific, and robust interactions that co-associate with *IFNG* gene expression using the RNA expression profiles of tumors from UCEC patients (source: TCGA) (Fig. 2a–c and Supplementary Fig. 2). Our analysis recapitulated the *SERPINB9* as one of the top five T-cell dysfunction signature genes carrying strong interactions with ICPs and associations with non-linear increases in IFNγ expression, as previously reported^3^ (Fig. 2d, e). Together with SERPINB9, we find *XCL1*, a gene encoding the chemokine secreted by activated CD8+ T-cells and natural killer cells^29^, and *CD5*, a gene for T-Cell receptor (TCR) established as a regulator of TCR and B-Cell Receptor (BCR) signaling^30^, to be most frequently associated with IFNγ levels through interactions with ICP genes.

Encouraged by these results, we explored other interactions within the network models of tumor-immune interactions in UCEC (Fig. 2d). We evaluated the association of each interacting gene pair in the networks with patient survival as assessed by *p* values from a log-rank statistical test. Among the 23 direct interactions, the co-expression of the CD70 and CD86 pair stood out as most associated with improved progression-free survival in UCEC patients (Fig. 2f, g). CD86 and CD70 are immune co-stimulatory proteins that are expressed in diverse immune cell types including activated lymphocytes and antigen-presenting cells^31,32^. Through interactions with their receptors (CTLA4, CD28, and CD27), both molecules promote T-cell activation and immune responses^18,31,32^.

Next, we asked whether the interaction between CD70 and CD86 is due to a co-expression in or co-association with identical immune cell types. Although both immune regulators are associated with overall immune infiltration (*R* = 0.55 and 0.64 for CD70 and CD86, respectively) as expected, we asked whether they are differentially associated with specific immune cell types. In the absence of comprehensive single cell omics data, we calculated the partial correlations between CD70 and CD86 genes with diverse immune cell type fractions based on CIBERSORT analysis of RNA expression profiles of tumors from endometrial cancer patients. This is an indirect measure of how each receptor engages with different immune cell types and does not provide definitive proof of their expression sites. We preferred the partial correlation metric as it enables elimination of confounding factors from other random variables while quantifying the direct association between two entities. CD86 is linked with macrophage fractions (both M1 and M2) as well as with regulatory T-cells and activated CD4+ memory T-cells, while CD70 is linked with regulatory T-cells and CD8+ T-cells (Supplementary Table 7). The partial correlation patterns for the two ICPs suggest differential associations with immune cell types and possible expression patterns in overlapping (likely in regulatory T-cells) but mostly distinct cell types including macrophages, CD4+ memory T-cells, and CD8+ T-cells. The nonuniform associations of CD70 and CD86 expression with different immune cell types suggest the interaction between the two immune stimulators is not an artifact of a uniformly affecting intrinsic tumor impurity manifested as immune infiltration or extrinsic impurity that may arise from sample collection (see ref. ^33^ for a detailed discussion of tumor impurity). This argument is also supported by our statistical validations that demonstrated a robust, specific, and significant interaction between the CD70 and CD86 compared to other immune checkpoints.

The specific (Q value < 0.1), robust (-log(*R*) > 0), significant (Q alue < 0.1), and high-ranked combined action scores suggest that expression of CD86 and CD70 co-associate with increased levels of IFNγ in tumors of UCEC patients (Fig. 2f). Moreover, we have observed that co-expression of CD70 and CD86 is associated with improved patient survival (Fig. 2g). The association with both IFNγ responses and patient survival suggests functional immune co-stimulatory roles for the CD86 and CD70 receptors in overlapping contexts within tumors of UCEC patients. Further studies, however, are needed to identify which CD86 and CD70 expression configurations and co-targeting strategies including agonists of both receptors as well as the addition of a third agent (e.g., an immune checkpoint inhibitor) may be tractable in the pre-clinical and clinical settings.

### T-cell dysfunction and immune checkpoint interactions in basal-like breast cancer

**Fig. 3:**
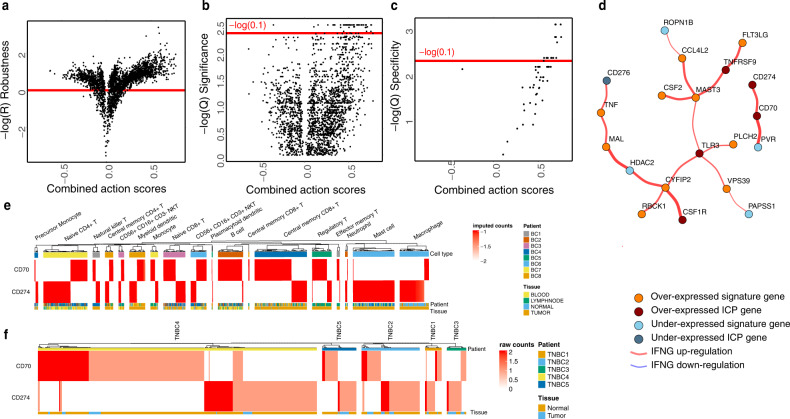
Interactions of T-cell dysfunction signature genes with immune checkpoints in breast cancer. ImogiMap-based assessment of combinatorial interactions for T-cell dysfunction signature genes in basal-like breast cancer (based on RNA expression data from tumors of 172 patients), and therapeutically actionable ICPs, based on their associations with the *IFNG* expression. **a** Combined action score robustness metric. A normalized root mean square deviation (RMSD) is calculated for each combined action score through random sampling (1000x) of sub-cohorts with 70% coverage of the complete cohort. Scores with high robustness, $-{{{{{\rm{log }}}}}}\left({{R}}\right)\, > \,0$ (red horizontal line) are selected for further analysis (see “Methods”). **b** The significance metric for the combined action score. An FDR (Benjamini-Hochberg (BH) method) corrected ${Q}$-value based on the Wilcoxon signed-rank test is calculated for robust scores. Scores with ${Q}_{\_{{{{{\rm{Significance}}}}}}}\, < \,0.1$ are selected for further analysis. **c** A BH-corrected *Q*-value for specificity is calculated for each robust and significant interaction. Scores with ${Q}_{\_{{{{{\rm{Specificity}}}}}}}\, < \,0.1$ are selected for further analysis. **d** The graphical network representing robust, significant, and specific combinatorial associations with IFNγ levels. Red (blue) edges represent upregulation (downregulation) of *IFNG* gene expression level. Dark red (dark blue) vertices identify overexpression (low-expression) of ICP genes and orange (blue) vertices identify overexpression (low-expression) of T-cell dysfunction signature genes. The single cell mRNA expression patterns of CD70 and CD274 in (**e**) breast cancer-associated immune cells^11^ and (**f**) tumors from five patients with triple-negative breast cancer patients^34^.

Through application of ImogiMap on RNA expression data from the TCGA basal-like breast cancer cohort (*N* = 172 patients) (TCGA, 2012), among 44 T-cell dysfunction signature, 29 immune checkpoints, and additional 20 genes that correspond to receptors or ligands of ICPs in CellPhoneDB database, we identified the significant, specific, and robust interactions that co-associate with *IFNG* gene expression (Fig. 3a–c and Supplementary Fig. 3, Supplementary Tables 1 and 5). We observed 18 statistically validated interactions (Fig. 3d) including the CD70 and CD274 pair which ranked among the top five pairs with the highest combined action scores. A negative interaction between high expression of CD70 and low expression of PVR is also noted. Other interacting pairs in the top five are CSF1R:CYFIP2, HDAC2:CYFIP2, and HDAC2:MAL.

As the inhibitors of PD-L1, which is encoded by the *CD274* gene, are already in clinical use and the gene product of *CD70* is another potentially interesting immunotherapy target, we focused on the CD274:CD70 interaction in the context of IFNγ expression in breast cancer. We addressed whether the interaction between CD70 and CD274 may be due to co-expression patterns in identical cell types. We used two sets of single cell RNA sequencing data^11,34^ to evaluate the mutual exclusivity of gene expressions in the breast cancer tumor microenvironment. We used imputed data from 45,000 immune cells from eight breast carcinoma patients^11^, and identified 600 cells that are CD70+ or CD274+ (Fig. 3e). The single cell RNA sequence analysis suggests that CD70 and CD274 are not co-expressed in identical immune cell types. CD70 is expressed in the CD8+ T-cells and B-cells while CD274 has higher expression in Mast cells, CD4+ T-cells, and macrophages compared to CD70 (Fig. 3). In addition, CD274 expression is higher in macrophages while CD70 is more abundant in regulatory T-cells (Fig. 3e). Both genes are expressed in similar degrees in CD4+ memory T cells. This is consistent with the partial correlation calculations using bulk RNA expression data (source: Breast Cancer TCGA) in which expression of CD274 is linked to the presence of activated and resting CD4+ memory T-cells, M1 macrophages as well as neutrophils, and expression of CD70 is correlated with activated CD4+ memory T-cells and regulatory T-cells (Supplementary Table 8). In a separate analysis, we used raw RNA-seq data from around 9000 cells originating from 5 TNBC patients^34^ and identified around 440 cells that were CD70+ or CD274+ (Fig. 3f) and confirmed lack of extensive co-expression in identical cells. Our single cell gene expression analysis suggests that CD274 and CD70 are not expressed in identical cells. The analysis, however, does not provide a direct measure of the average expression of each checkpoint in cell types as we analyzed only the cells expressing one of the checkpoints (CD274, CD70) and did not account for total number of cells within each cell type. Our observations showed that CD70 and CD274 are co-associated with IFNγ expression and are predominantly expressed in different immune cell types within the tumor microenvironment.

## Discussion

The ImogiMap statistical framework aims to extract the functional immune checkpoints that engage with the tumor ecosystems and modulate the tumor and immune characteristics. We have implemented an integrated approach based on the stratification of patient groups, calculation of a combined action score that quantifies the co-associations of ICP and TAP genes followed by comprehensive statistical validation and network representation. We have also provided an R-package and web interface to facilitate future applications of ImogiMap.

Diverse methods for inference of biological interactions (e.g., signaling interactions, oncogenic co-alterations, and immune relations) have been implemented by our and other groups, a few examples being pairwise or partial correlations, database-driven informatics approaches, regression models, ordinary differential equations, stochastic gradient descent for predictive machine learning models and more recently deep learning approaches^35–40^. These methods provide varying advantages such as quantitative predictions of responses to previously untested perturbations in individual samples or building interaction models that are less affected by confounding factors. The ImogiMap scheme provides a unique set of advantages. First, ImogiMap can be applied with sparse data as it relies on the calculation of pairwise scores for immune and tumor processes. This is an important feature as sparse data is common in translational and clinical settings, limiting the implementation of sophisticated machine learning methods. Second, the method does not require rich drug perturbation response or temporal data. Although the use of perturbational and temporal constraints may better enable detection of likely causal interactions, the baseline datasets are still able to capture statistically validated associations. Third, the stratification of patients may enable the selection of relevant patient sub-cohorts based on the co-associations of immune checkpoints and tumor-related events. Such stratification and patient sub-cohort selection may be highly useful in precision oncology applications while partial correlation- or regression-based methods do not immediately lead to a feasible strategy for patient selection. The relatively simple implementation of ImogiMap also enables the incorporation of versatile combined action scores as well as statistical validation schemes. In conclusion, the ImogiMap method is a simple, versatile, and yet informative method for quantitative characterization and statistical validation of the higher-order interactions between oncogenic and immune events.

The flexibility and statistical rigor of the ImogiMap algorithm may enable generation of hypotheses for the discovery of previously unreported physical or functional interactions. Our current implementation is optimized for immune interactions partly due to the immediate need in the field. ImogiMap, however, is not necessarily limited to tumor-immune interactions, and with its flexibility, it enables different modes of discovery. First, the algorithm can be applied to identify diverse mediators that may together impact a given phenotype (predict interacting genes that associate with a particular phenotype). Execution of ImogiMap with a large gene set against a phenotype of interest may generate a list of interactions that are significantly co-associated with the target phenotype. Second, the algorithm can be used to discover novel interaction partners of a gene, whose impact on a given phenotype is already known (discovery of additional regulators of a phenotype with already known mediators). In this case, the ImogiMap can be executed for a pre-defined gene against a large set of candidate genes and the outcomes may guide selection of interactions that mediate a particular phenotype. A major challenge in ImogiMap is selection of likely functional events based on co-associations, which do not dictate causation. This is particularly challenging when baseline, correlative data is used from large patient cohorts as in the case of TCGA datasets. A potential solution to select likely causal interactions is to use time series and perturbation response data. The sequence of expression vs. phenotypic events in time or differential response to perturbations may provide the constraints to establish causality and directionality of interactions. Indeed, the incorporation of such data into ImogiMap is straightforward with minimal design differences. Experimental validation of the predictions, however, is essential to claim a novel discovery regardless of the underlying data modality as well as time and perturbation schemes.

Various immune-oncology bioinformatics and deconvolution algorithms such as CIBERSORT and Kassandra have enabled the estimation of the immune decomposition of tumor ecosystems with increasing accuracy^41,42^. However, such methods do not capture the higher-level interactions between immune and tumor compartments. As a strong and highly popular tool, TIMER and its web interface provide a way to analysis of immune-tumor interactions^14^. The TIMER algorithm and web interface enable researchers to explore the immune composition of tumor microenvironments through the use of established immune deconvolution algorithms and compute the correlations between oncogenic alterations with immune cell fractions and phenotypic outcomes (e.g., survival). ImogiMap provides a series of advantages over the existing computational approaches. First, the ImogiMap is a statistically rigorous algorithm beyond correlation analyses for the detection of oncogenic alterations and immune checkpoints with an emphasis on therapeutically actionable immune processes. Second, the stratification of patient cohorts based on the co-expression patterns enables not only the detection of interactions but also the establishment of a framework for patient selection in precision oncology applications. Third, the method captures tumor-immune interactions in the context of diverse immune-associated phenotypes such as immune infiltration, immune cell types, IFNγ expression, or inflammation signatures. Fourth, the method incorporates receptor-ligand interactions to assess the tumor-immune interface. Finally, the ImogiMap is highly flexible and enables incorporation of new oncogenic alteration types, immune checkpoints as well as phenotypes.

Our findings suggest that ImogiMap generates hypotheses on (and confirms previously reported) relations between tumor-associated processes and immune regulation at a scale not accessible easily by experimental methods. ImogiMap may help basic and translational researchers to discover novel immune-tumor interactions and potential vulnerabilities to combination therapies that target the immune-tumor interactions. The outcomes of ImogiMap analyses may improve the repertoire of actionable ICP targets, and identify patient cohorts that may respond to combination therapies based on their molecular signatures.

## Methods

### Immune checkpoints (ICPs) and immune-associated phenotypes (IAPs)

Genes for 14 actionable ICPs as well as 15 additional genes of corresponding ligands or receptors are included (Supplementary Table 1) based on a literature search and recommendations by the CRI clinical accelerator team^18,43–47^. Users have the option to input their own curated list of ICPs based on custom mRNA expression data, proteomics, or quantitative histologic assessments (e.g., IHC staining and quantification with relevant markers). In the absence of user-provided data, ImogiMap uses API functions in the curatedTCGAData package (Bioconductor), to access TCGA mRNA data. ImogiMap includes a list of 29 immune-associated phenotypes (Supplementary Tables 2 and 3). Levels of vascularization, EMT, T-cell inflamed, and IFNG RNA expression are each calculated using their corresponding signature gene lists^16,21–23^. Each phenotype score is quantified as the mean of *z*-values of log-scaled mRNA expression levels of the genes in the phenotype signature. For TCGA-based analysis, precalculated values of immune cell infiltration and fractions of immune cell types (Supplementary Table 3) are implemented based on DNA methylation^18^ and CIBERSORT^48^ assessments, respectively. Quantification methods, sources, and signature genes for immune phenotypes are listed in Supplementary Table 2. Users have the option to include their own IAP values based on custom (omics or histology) data or TCGA data.

### IAP normalization and scaling

The measurements of IAPs are rescaled and normalized using a logistic sigmoidal function. The scaling facilitates comparisons of phenotypes and resulting combined action scores across different phenotypes. The rescaling is formulated to transform IAPs to a range of [0,1]. Three separate rescaling functions are formulated based on the initial range of IAP values across samples:

1 $${x}_{i,{{{{{\rm{IAP}}}}}}}^{{{{{{\rm{scaled}}}}}}}=\left\{\begin{array}{ll}{x}_{i,{{{{{\rm{IAP}}}}}}}, & \forall \,{{{{{\rm{IAP}}}}}}\ni \forall {x}_{i,{{{{{\rm{IAP}}}}}}}\in [0,1]\\ \frac{\tanh \left(\frac{{x}_{i,{{{{{\rm{IAP}}}}}}}}{{\sigma }_{{x}_{{{{{{\rm{IAP}}}}}}}}}\right)+1}{2},\, & \forall \,{{{{{\rm{IAP}}}}}}\ni \forall {x}_{i,{{{{{\rm{IAP}}}}}}}\in [-\infty ,\infty ]\,\\ {\tanh} (\frac{{x}_{i,{{{{{\rm{IAP}}}}}}}}{{\sigma }_{{x}_{{{{{{\rm{IAP}}}}}}}}}), & \forall \,{{{{{\rm{IAP}}}}}}\ni \forall {x}_{i,{{{{{\rm{IAP}}}}}}}\in [0,\infty ]\end{array}\right.$$

where *i* is the sample index, IAP denotes a particular immune-associated phenotype, *x*_*i*,IAP_ is the readout of the phenotype for sample *i*, *σ*_*X*,IAP_ is the standard deviation of readouts for IAP over all samples. The resulting *x*_*i*,AIP_^scaled^ has a dynamic range of [0,1] for any IAP and therefore cross-comparisons of metrics (e.g., combined action scores) calculated from scaled IAP values are possible. To demonstrate how the mathematical transformations affect phenotypes, the distribution of phenotypes before and after normalization is depicted in Supplementary Fig. 4.

### Calculation of gene pair combined action scores on IAPs

The combined action scores between the immune checkpoint and tumor-associated genes are calculated based on the non-linear deviation of the observed IAPs from the expected values for the condition that two genes are independently associated with the IAP. To measure combined action scores that quantify tumor-immune interactions, samples are first stratified into four groups based on median expression levels of two genes, as explained in the main text (i.e., “LL”, “LH”, “HL”, “HH”). First, to calculate a combined action score between two genes, a “baseline” sub-cohort and a target sub-cohort, which carry the presumably least and most co-associated IAP level respectively, are selected among the LL, LH, HL, or HH sub-cohorts. In the absence of a priori assumption on a relationship between the expression of single genes and IAP levels (i.e., IAP-activating vs. IAP-deactivating genes), we analyze all possible configurations such that either the two genes may be activating/deactivating or have opposing relations with the IAP. For each configuration, the relevant baseline and target sub-cohorts with contrasting gene expression levels are identified and a combined action score is calculated as explained below. The configuration which results in the highest combined action score is selected as the metric for the potential combinatorial impact of the two genes on the IAP and is further analyzed.

The combined action score is quantified as follows: first, for each sub-cohort, the median deviation of IAP, ${M}_{n}$, from the baseline is quantified as:

2 $${M}_{n}={{{{{\rm{med}}}}}}({x}_{i,{{{{{{\rm{IAP}}}}}}}}^{{{{{{{\rm{scaled}}}}}}}})-{{{{{\rm{med}}}}}}\left({{x}_{i,{{{{{{\rm{IAP}}}}}}}}^{{{{{{{\rm{scaled}}}}}}}}}_{{{{{{{\rm{Baseline}}}}}}}}\right).$$

To reduce noise and ensure that the sign of ${M}_{n}$ is correctly estimated, ${M}$ values that are smaller than their corresponding standard error of the median, ${{{{{\rm{sem}}}}}}(M)$, are set to zero:

3 $${M}_{n}=\left\{\begin{array}{c}{M}_{n},\,{{{{{\rm{abs}}}}}}({M}_{n}) \, > \, {{{{{\rm{sem}}}}}}({M}_{n})\\ 0,\,{{{{{\rm{abs}}}}}}({M}_{n}) \, < \, {{{{{\rm{sem}}}}}}({M}_{n})\end{array}\right.,$$

in which the standard errors are calculated using standard errors within each sub-cohort as:

4 $${{{{{\rm{sem}}}}}}\left({M}_{n}\right)=\root {2} \of {{{{{{{{\rm{sem}}}}}}}}_{n}^{2}+{{{{{{{\rm{sem}}}}}}}}_{{{{{{{\rm{Baseline}}}}}}}}^{2}}.$$

${M}_{E}$, an expected value for *M*_*N*_ in the target group, is calculated under the condition that each gene acts independently on the IAP. This is achieved through the use of either of the two reference additivity models that are implemented in ImogiMap, the Bliss independence model^25^ or the Highest Single Agent model^24^. Under each model ${M}_{E}$ is defined as:

5 $${M}_{E}=\left\{\begin{array}{c}{{{{{\rm{max }}}}}}\left({M}_{1},{M}_{2}\right)\,{{{{{{\rm{Highest}}}}}}}\,{{{{{{\rm{Single}}}}}}}\,{{{{{{\rm{Agent}}}}}}}\,{{{{{{\rm{model}}}}}}}\\ {M}_{1}+\,{M}_{2}-{M}_{1} \times {M}_{2}\,{{{{{{\rm{Bliss}}}}}}}\,{{{{{{\rm{independece}}}}}}}\,{{{{{{\rm{models}}}}}}}\end{array}\right.$$

In which ${M}_{1}$ and ${M}_{2}$ are calculated median values from Eq. (3) for the two remaining sub-cohorts that are not baseline or target. We define a combined action score, $S$, as the difference between the measured and the expected ${M}$ value in the target sub-cohort:

6 $$S=\left\{\begin{array}{ll}{{{{{\rm{sign}}}}}}(M)\times ({M}_{{{{{{\rm{target}}}}}}}-{M}_{E}), & {M}_{{{{{{\rm{target}}}}}}}-{M}_{E} \, > \, 0\\ \hfill 0, & {M}_{{{{{{\rm{target}}}}}}}-{M}_{E} \,\le\, 0\end{array}\right.$$

in which ${{{{{{\rm{sign}}}}}}}(M)$ is the direction of the change from the baseline in all sub-cohorts. Note that in the reference additivity models ${M}_{E}$ for the target group can only be estimated if all three ${M}_{n}$ values have the same sign, which indicates the same direction of change from baseline. If ${{{{{{\rm{sign}}}}}}}(M)$ differs in different sub-cohorts, no combined action score will be calculated, and a missing value will be reported. Finally, the interactions with high combined action scores (${M}_{{{{{{{\rm{target}}}}}}}}-{M}_{E} \, > \, 0$) are selected for further statistical evaluation.

### Statistical evaluation of tumor-immune interactions

Through statistical assessment of the combined action scores between tumor-associated and immunoregulatory genes, we filter out the scores that are not robust, statistically significant, or specific.

“Robustness of interactions” to the underlying data configuration is tested through bootstrapping (1000x) of samples with partial coverage (default is 70% of a complete cohort), re-stratifying patient sub-cohorts based on the bootstrapped data, and calculating the combined action scores. The robustness score is defined as the normalized root-mean-square deviation of combined action scores between complete, ${S}_{{{{{{{\rm{complete}}}}}}}}$, and partial, ${S}_{{{{{{{\rm{partial}}}}}}}}$, datasets:

7 $$R=\frac{\root {2} \of {\frac{{{\sum }_{i}^{n}({S}_{{{{{{{\rm{complete}}}}}}}}-{S}_{{{{{{{\rm{partial}}}}}}}}^{i})}^{2}}{n}}}{{{{{{\rm{abs}}}}}}({S}_{{{{{{{\rm{complete}}}}}}}})}$$

where $S$ is the combined action score, and $n$ is the number of sampling to determine the ${S}_{{{{{{{\rm{partial}}}}}}}}$ set. Normalization with respect to the absolute value of ${S}_{{{{{{{\rm{complete}}}}}}}}$ serves as the scaling factor to enable comparison of *R* values across diverse interactions with high combined action scores. The least sensitive to the exact data configuration, therefore more robust and reliable scores, have lower $R$ and are assessed by ranking the sensitivity scores for all interactions.

“The statistical significance of interactions” is assessed with a Wilcoxon signed-rank sum test comparing the rankings of immune phenotype levels in the target sub-cohort and the remaining three sub-cohorts (LL, LH, HL, HH). In the statistical assessment, the null model is that the immune phenotype levels in the target sub-cohort and at least one of the remaining three sub-cohorts are sampled from the same distribution. The maximum of the three Wilcoxon *p* values is reported as the *p* value of the interaction. The sub-cohort-specific IAP values are compared on boxplots that contain Wilcoxon *p* values (BH-corrected for multiple hypothesis testing) for group comparisons.

“The specificity of an interaction” between a TAP and ICP gene pair is quantified. The specificity implies that the observed combined action score between the two genes of interest is significantly higher than the combined action scores of each of the genes in the pair against other genes in the genome. First, two separate null models are generated by calculating the combined action scores of each of the two genes against a set of genes that are randomly sampled from the genome (default ${N}_{{{{{{{\rm{genes}}}}}}}}=1000$). For each of the two genes, a *p* value is calculated against the two null models. The highest of the two *p* values, ${p}_{\max }$, which indicates the lowest specificity is used to assess the specificity of the interaction against the whole genome. A low *p* value (${p}_{\max } \, < \, 0.05$) indicates high specificity. The *p* values are corrected for multiple hypothesis testing using the BH-method. 

### Network models of immune-tumor interactions

The interactions that are significantly strong (high combined action score), robust and specific are selected to construct a network model which captures the combinatorial interactions between the tumor-associated and immune genes. In the network models, the edges represent interactions between gene pairs for which a significant, specific, and robust combined action score on IAP exists. Weak interactions are also filtered out based on a user-defined cut-off value. The network model is visualized using the igraph application^49,50^.

### Ligand-receptor interactions

To interpret interactions from network models, it is important to know potentially relevant ligand-receptor interactions in the tumor microenvironment as they are the key components in mediating cell-cell interactions. To integrate ligand-receptor interactions, we use annotations in the CellPhoneDB database to infer the gene pairs corresponding to protein–protein interactions. Given input gene lists, ImogiMap automatically searches the CellPhoneDB database for potential ligand-receptor pairs and adds them to the original gene lists. Combined action scores are computed for all gene pairs. For each gene pair, the output will include a combined action score, statistical analysis, and whether the gene pair is an inferred ligand-receptor pair in the CellPhoneDB database. The receptor-ligand interactions are annotated on the network models.

### Partial correlations

In each cohort, the partial correlation between expression of a gene and fraction of immune cell types are calculated using TCGA RNASeq2GeneNorm data and CIBERSORT cell type fractions correspondingly. For each immune cell type, we used R ppcor library to calculate the Spearman partial correlation of the gene-immune cell type while controlling for the rest of the immune cell types. The resulting *p* values are adjusted for multiple hypothesis testing using the Bonferroni method.

### Statistics and reproducibility

All statistical analyses are described in detail in the “Statistical evaluation of tumor-immune interactions” section and the main text. To ensure reproducibility, the source code is provided on GitHub (see “Data and code availability section”) and we used publicly available TCGA datasets.

### Reporting summary

Further information on research design is available in the Nature Portfolio Reporting Summary linked to this article.

## Supplementary information

Korkut_Peer Review File

Supplementary Information

Reporting Summary

## Data Availability

The data used in the manuscript are publicly available. Numerical source data for Figs. 2 and 3 are included in the github repository (https://github.com/korkutlab/imogimap/tree/master/inst/Tcell_dysfunction_analysis) along with the code (analysis.r) to re-create the plots. Same code generates numerical source data for Supplementary figures and creates the Supplementary figures.

## Code availability

ImogiMap R package and detailed user guide are available at https://github.com/korkutlab/Imogimap. Web interface is available at https://bioinformatics.mdanderson.org/apps/imogimap.
