# Supplementary material for: Mapping the functional interactions at the tumor-immune checkpoint interface
Source: Commun Biol. 2023 Apr 27;6:462. doi: 10.1038/s42003-023-04777-3 (PMC10140040; doi:10.1038/s42003-023-04777-3)
Supplement: Supplementary file 2 — Supplementary Information [file 42003_2023_4777_MOESM2_ESM.pdf]

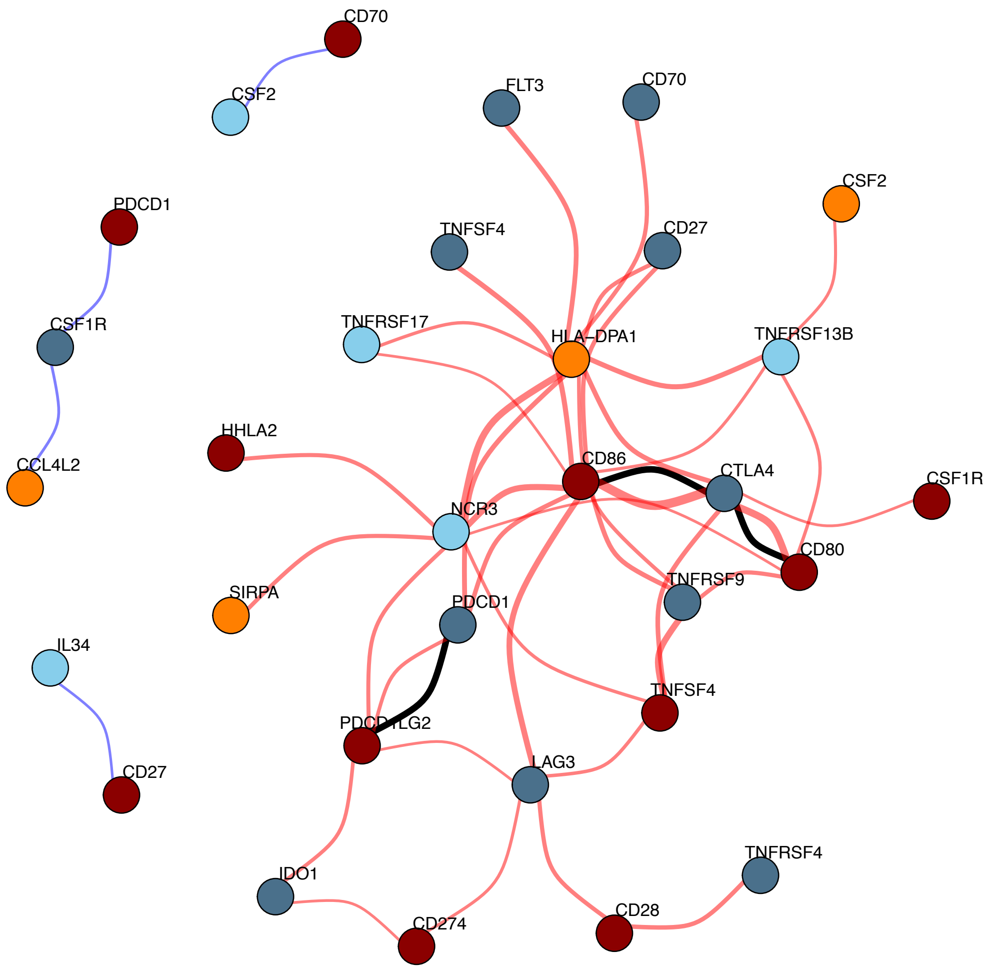

Supplementary ***Figure 1.*** *A Sample graphical network depicting receptor ligand pairs from CellphoneDB as black edges. Red/Blue edges depict combined action scores of immune checkpoints and their corresponding ligand/receptors on fraction of M2 macrophages (IAP) in TCGA LUAD samples (576 samples). The receptor ligand pairs which are also inferred as adjacent vertices, namely PDCD1 and PDCD1L2, CTLA4 and CD80, CTLA4 and CD86, are represented with black edges.*

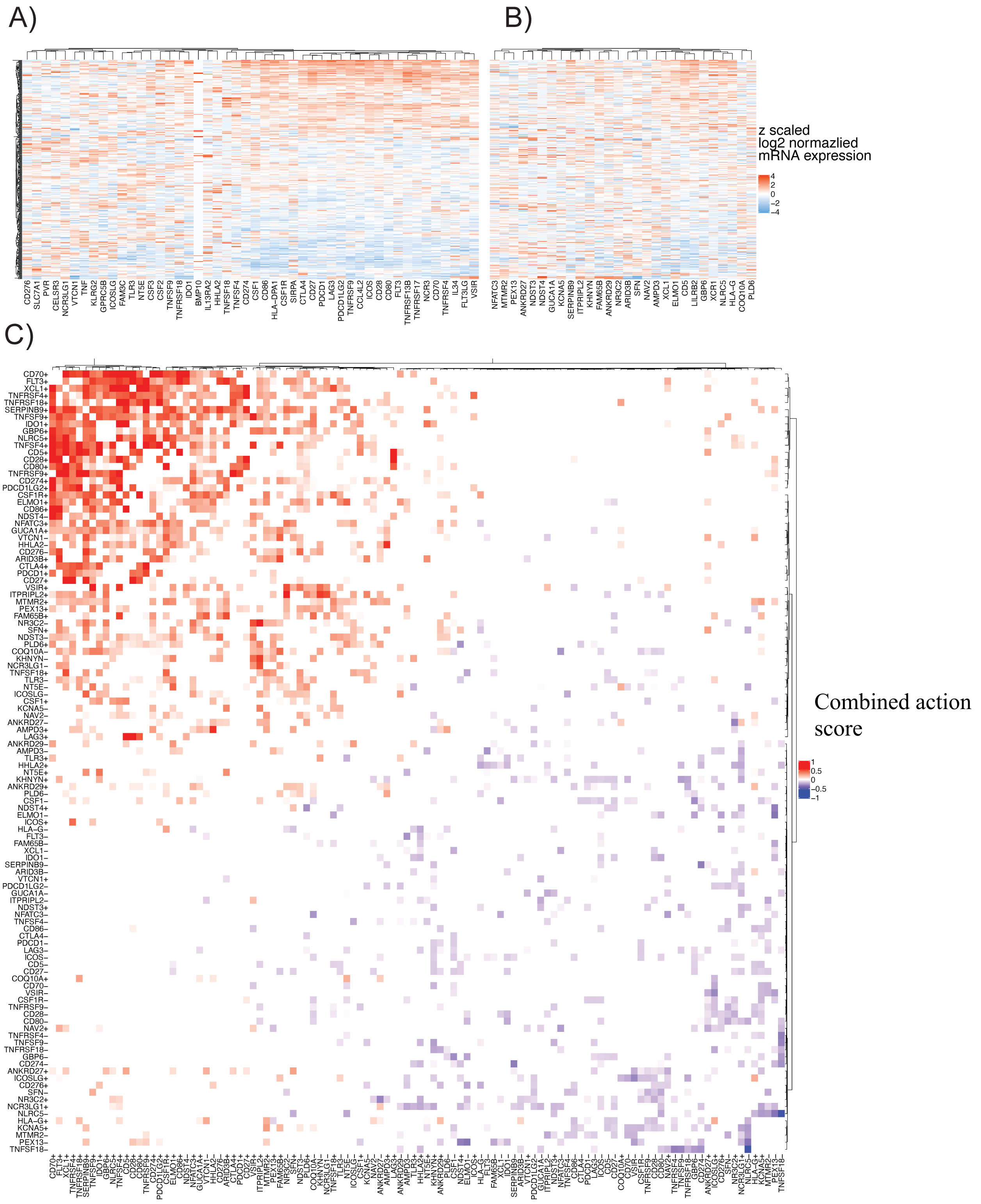

Supplementary *Figure 2.* *A. mRNA expression of ICP genes in UCEC. B. mRNA expression of the T-cell dysfunction signature for UCEC, that serve as the tumor associated process (TAP) genes in this application. C. The landscape of interactions quantified by combined action scores in UCEC between the ICP and TAP genes for co-association with the IFNG levels. The positive (red) and negative (blue) interactions correspond to IFNG increases and decreases, respectively.*

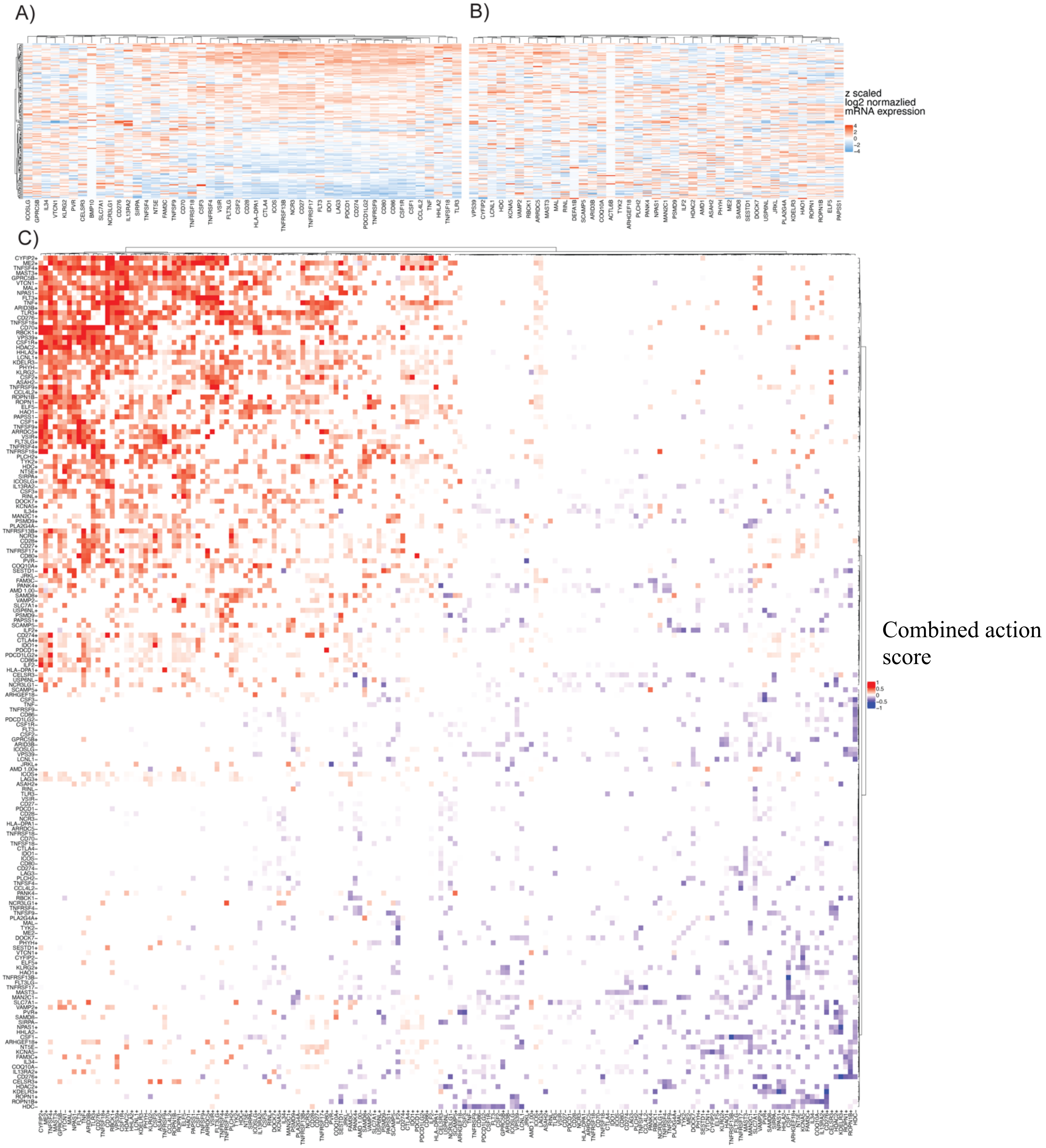

Supplementary *Figure 3.* *A. mRNA expression of ICP genes in basal-like breast cancers. B. mRNA expression of the T-cell dysfunction signature (tumor associated process (TAP)) genes for basal-like breast cancers. C. The landscape of interactions quantified by combined action scores between the ICP and TAP genes for co-association with the IFNG levels. The positive (red) and negative (blue) interactions correspond to IFNG increases and decreases, respectively.*

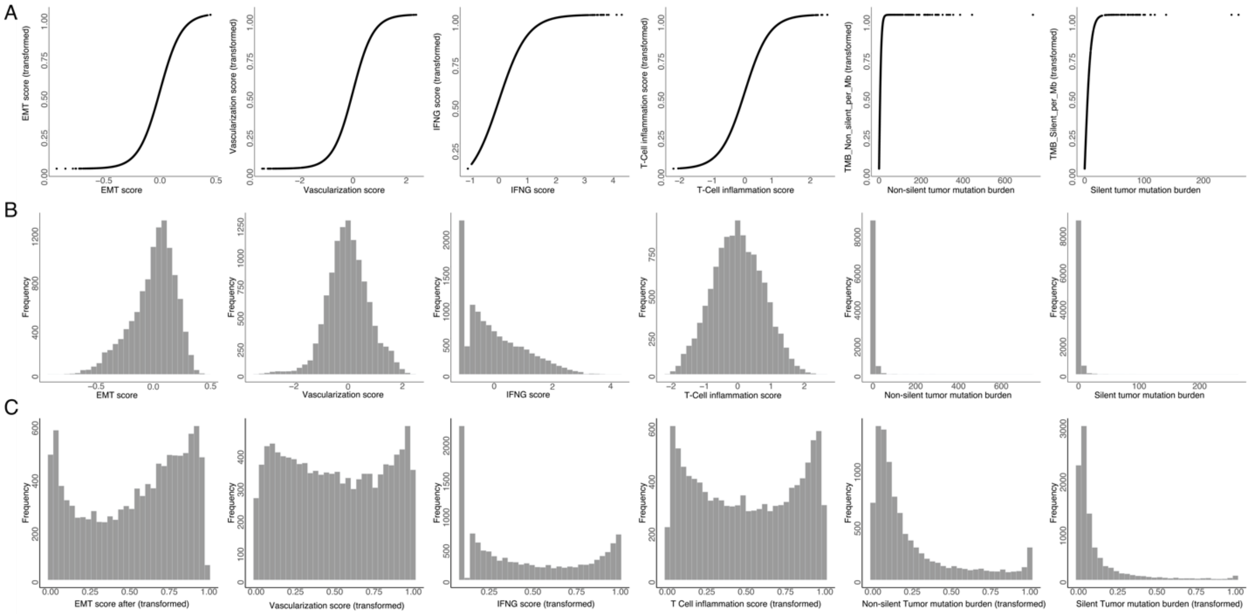

Supplementary *Figure 4.* *a) Graphs of IAP transformation functions. b) Distribution of IAP values in PanCan TCGA data. c) Corresponding distribution histogram of transformed IAP through execution of equation 1. Note that Leukocyte fraction and fractions of immune cell types do not change under transformation, as they are inherently positive and smaller than 1.*

Supplementary Tables

Supplementary Table 1. Immune checkpoints and their ligands or receptors.

Gene name	Protein name	Potential interactions
PDCD1	PD-1	PD-L1, PDL2
CD274	PD-L1	PD-1
CTLA4	CTLA-4	CD28, B7-1, B7-2
CSF1R	CSF1R	CSF1
TNFRSF9	4-1BB	4-1BBL
TNFRSF18	GITR	GITRL
CD27	CD27	CD70
ICOS	ICOS	B7-h2
TNFRSF4	OX40	OX40LG
PDCD1LG2	PD-L2	PD-1
CD70	CD70	CD27
CSF1	CSF1	CSF1R
TNFSF9	4-1BBL	4-1BB
TNFSF4	OX40LG	OX40
TNFSF18	GITRL	GITR
CD28	CD28	CTLA-4
CD80	B7-1	CTLA-4
CD86	B7-2	CTLA-4
ICOSLG	B7-h2	ICOS
CD276	B7-h3	
VTCN1	B7-h4	
VSIR	B7-h5	
NCR3LG1	B7-h6	
HHLA2	B7-h7	

LAG3	LAG-3	
IDO1	IDO1	
FLT3	FLT3	
NT5E	CD73	
TLR3	TLR-3	

Supplementary Table 2. ImogiMap's default immune-associated phenotypes

**Phenotype**	**Source**
EMT score	mRNA expression of EMT signature genes (Mak et al., 2016)
Vascularization	mRNA expression of Angiogenesis signature genes (Masiero et al., 2013)
T cell Inflammation	mRNA expression of T cell inflamed signature genes (Ayers et al., 2017)
IFNG score	mRNA expression of *IFNG* (Gao et al., 2016)
Non-silent tumor mutation burden per MB	Whole exome or genome sequencing (Hoadley et al. 2018)
Silent tumor mutation burden per MB	Whole exome or genome sequencing (Hoadley et al. 2018)
Leukocyte fraction	DNA methylation (Hoadley et al. 2018)
22 immune cell type fractions	CIBERSORT analysis of mRNA expression (Thorsson et al. 2018)

Supplementary Table 3. 22 immune cell types from CIBERSORT analysis

Memory B Cells
Naive B Cells
Plasma Cells
CD8 T cells
Naive CD4 T cells
Activated memory CD4 T cells
Resting Memory CD4 T cells
Follicular Helper T Cells
Regulatory T Cells
Gamma Delta T Cells
Activated Natural killer Cells
Resting Natural Killer Cells
Monocytes
M0 Macrophages
M1 Macrophages
M2 Macrophages
Resting Dendritic Cells
Activated Dendritic Cells
Activated Mast Cells
Resting Mast Cells
Eosinophils
Neutrophils

Supplementary Table 4. T-cell dysfunction signature genes for UCEC (Jiang et al., 2018)

AMPD3
ANKRD27
ANKRD29
ARID3B
CD5
COQ10A
ELMO1
FAM65B
GBP6
GUCA1A
HLA-G
ITPRIPL2
KCNA5
KHNYN
MTMR2
NAV2
NDST3
NDST4
NFATC3
NLRC5
NR3C2
PEX13
PLD6
SERPINB9
SFN
XCL1

Supplementary Table 5. List of T-cell dysfunction signature genes for TNBC (Jiang et al., 2018)

ARID3B
SCAMP5
ARRDC5
NPAS1
VPS39
ACTL6B
KCNA5
MAST3
ARHGEF18
LCNL1
VAMP2
RBCK1
TYK2
TTTY13
PLA2G4B
MAN2C1
LOC285095
RINL
CYFIP2
ASAH2
PLCH2
MAL
COQ10A
PSMD9
PANK4
HDC
KDELR3
ROPN1B
ME2
HDAC2
HAO1
SAMD8

ROPN1
ILF2
DEFA1B
SESTD1
AMD 1.00
PHYH
JRKL
PAPSS1
USP6NL
PLA2G4A
DOCK7
ELF5

Supplementary Table 6. Ligand-receptor pairs for ICP and T-cell dysfunction genes based on CellPhoneDB database.

Gene1	Gene2
CD27	CD70
PDCD1	CD274
PDCD1	PDCD1LG2
CD80	CD274
CD28	CD80
CD28	CD86
CTLA4	CD80
CTLA4	CD86
CTLA4	CD28
CSF1R	CSF1
CSF1R	IL34
CSF1R	CSF3
CSF1	SLC7A1
CSF1	SIRPA
CSF1	CELSR3
CSF2	CSF1R
NCR3	NCR3LG1
FLT3	FLT3LG
ICOSLG	ICOS
TNFSF18	TNFRSF18
TNFSF4	TNFRSF4
TNFSF9	TNFRSF9
BMP10	VSIR
CCL4L2	VSIR
TNF	VSIR
TNFRSF13B	CD70
CD70	TNFRSF17
CD70	GPRC5B
TNF	ICOS
PDCD1	FAM3C

KLRG2	TNFSF9
HLA-DPA1	TNFSF9
PVR	TNFSF9
TNFSF9	IL13RA2
TNFSF9	ADGRG5
HLA-G	LILRB2
XCL1	XCR1
XCL1	ADGRV1

Supplementary Table 7. Partial (spearman) correlation values of CD86 and CD70 with immune cell types in UCEC patients. For each gene-immune cell type pairwise partial correlation is calculated in the presence of other immune cell types.

gene	Immune cell types	Partial correlation	Pvalue(FDR adjusted)
CD86	Macrophages.M2	0.272	6.97e-06
CD86	Macrophages.M1	0.215	1.34e-03
CD86	T.Cells.Regulatory.Tregs	0.215	1.36e-03
CD86	T.Cells.CD4.Memory.Activated	0.200	4.51e-03

gene	Immune cell types	Partial correlation	Pvalue (FDR-adjusted)
CD70	T.Cells.CD8	0.189	9.76e-03
CD70	T.Cells.Regulatory.Tregs	0.172	3.17e-02

Supplementary Table 8. Partial (spearman) correlation values of CD70 and CD274 in TNBC patients. For each gene-immune cell type pairwise partial correlation is calculated given other immune cell types.

gene	Immune cell types	Partial correlation	Pvalue(FDR adjusted)
CD274	T.Cells.CD4.Memory.Activated	0.477	1.61e-08
CD274	Macrophages.M1	0.431	8.33e-07
CD274	T.Cells.CD4.Memory.Resting	0.326	9.88e-04
CD274	Neutrophils	0.244	6.06e-02

gene	Immune cell types	Partial correlation	Pvalue (FDR-adjusted)
CD70	T.Cells.CD4.Memory.Activated	0.289	7.90e-3
CD70	T.Cells.Regulatory.Tregs	0.239	7.54e-2
