# Supplementary material for: Mapping the functional interactions at the tumor-immune checkpoint interface
Source: Commun Biol. 2023 Apr 27;6:462. doi: 10.1038/s42003-023-04777-3 (PMC10140040; doi:10.1038/s42003-023-04777-3)
Supplement: Supplementary file 1 — Korkut_Peer Review File [file 42003_2023_4777_MOESM1_ESM.pdf]

nature portfolio

Peer Review File

Mapping the functional interactions at the tumor-immune checkpoint interface.

Open Access This file is licensed under a Creative Commons Attribution 4.0 International License, which permits use, sharing, adaptation, distribution and reproduction in any medium or format, as long as you give appropriate credit to the original author(s) and the source, provide a link to the Creative Commons license, and indicate if changes were made. In the cases where the authors are anonymous, such as is the case for the reports of anonymous peer reviewers, author attribution should be to 'Anonymous Referee' followed by a clear attribution to the source work. The images or other third party material in this file are included in the article's Creative Commons license, unless indicated otherwise in a credit line to the material. If material is not included in the article's Creative Commons license and your intended use is not permitted by statutory regulation or exceeds the permitted use, you will need to obtain permission directly from the copyright holder. To view a copy of this license, visit http://creativecommons.org/licenses/by/4.0/.

Reviewers' comments:

Reviewer #1 (Remarks to the Author):

In this manuscript, Bozorgui et al. introduce ImogiMap, a method for identifying synergies between tumor-associated processes (TAPs) and immune checkpoint genes (ICPs). This is achieved through co-association with immune-associated processes (IAPs). While ICPs are typical surface proteins expressed by tumor cells and immune cells, TAPs and IAPs are broadly defined as genes involved in cancer and immunogenicity.

The algorithm first divides samples based on expression of the inquiry ICP gene and TAP gene, and calculates a synergy score between the two based on the deviation of an IAP to its baseline. Statistically, this can be done much more elegantly through condition correlation, or a simple regression model accounting for both TAP and IAP levels, assuming ICP gene expression level is the response variable.

A more critical issue is data interpretation. In their example (Fig 2D), they show ICP genes are overexpressed, and they suggest CD70 and CD86 have synergistic interaction. However, both genes are highly expressed by APCs, and their "interaction" could simply reflect their co-expression by the same set of immune cells. In fact, all the ICP genes in this example show high expression, suggesting a common covariate, which I suspect is tumor purity.

The manuscript did not clearly explain why engaging IAP helps find synergy between ICP and TAP genes. In practice, most of the IAP features are just immune cell abundances predicted by CiberSort. The definition of IAP is obscure. For instance, EMT is considered an IAP. If this is based on the literature, there should be many more pathways/processes that have been associated with tumor immunity.

A minor issue is the exaggerated wording. I am not convinced this tool can "inform on underlying mechanisms that jointly relate TAPs to … ICPs and IAPs," nor provide "mechanistic links between tumors and the immune system." I suggest the authors to tone down their wording.

Reviewer #2 (Remarks to the Author):

In this interesting paper, the authors proposed a statistical framework, termed ImogiMap to quantify the tumor-immune interactions from gene expression data. ImogiMap automates the combinatorial searches for interactions between tumor-associated processes and immune checkpoint processes (TAP and ICP) based on their synergistic co-associations with immune-associated phenotypes (IAP) of interest. The input to ImogiMap is the mRNA expression data for ICP and TAP genes, and data (e.g., RNAseq, microarray, or histological) that quantify IAP levels from the matched samples. The statistic analysis implemented in the paper is reasonable and convincing. Each interaction is statistically evaluated for robustness, statistical significance, and specificity.
It is reassuring to see that this study can re-identify the known synergistic interaction between SERPINB9 and CTLA4, and their associations with nonlinear increases in IFNG expression. This offers the mechanics explanation that high expression of SERPINB9 confers resistance to CTLA4 checkpoint inhibition. It also justifies the therapeutic benefit from co-targeting SERPINB9 and CTLA4.
This is a timely study filling an unexplored field. ImogiMap can help basic and translational researchers to discover novel immune-tumor interactions at a scale not accessible easily by experimental methods.
Some minor issues:
1）From the paper in figure 2G, the high expression of both CD86 and CD70 is associated with better survival in UCEC patients. It is confusing to read "These findings posit the combinatorial targeting of CD86 and CD70 with agonist agents as a new candidate for immunotherapy with likely improved responses in UCEC patients whose tumors carry high CD86 and CD70 expression. ". Does the author actually propose this gene pair as new candidates for immunotherapy for UCEC

patients whose tumors carry LOW CD86 and CD70 expression?
2）To inform on the underlying mechanisms, it will be a good idea to strengthen the network visualization by incorporating additional network informatics tools (e.g., protein-protein interaction, ligand-receptor interaction) into your network models as an option for users to visualize/analyze the network.

Reviewer #3 (Remarks to the Author):

The authors (Bozorgui et al.) developed a method and computational tool (ImogiMap) to identify the interactions of tumor associated processes (TAP) and immune associated processes or phenotypes (IAP) such as estimated tumor infiltrated lymphocytes (TILS) or IFNG signaling via immune checkpoints and ligands (ICP). I'm intrigued by the presented method including synergy scores and their statistical evaluation, whereby samples (patients) are divided based on the median expression levels of a TAP-ICP gene pair into 4 groups (LL,LH,HL,HH) and their activating/deactivating effect on the IAP (scaled to [0,1]) is evaluated borrowing concepts as used for studying drug combinations.
The method is statistical sound and well explained, the implementation in R is in principal working (had a few configuration problems) and the manuscript is well written.
The authors provided also an worked example with a previously determined T cell dsyfuntion signature (Jiang et al. 2018) and demonstrated that the most outstanding gene of these signature, SERPINB9, together with the immune checkpoint CTLA4 in the endometrial cancer TCGA cohort (UCEC) is associated with the IFNG response. This demonstrats, that the tool is very flexible and not only oncogenes can be used but also other gene lists, which are not limited to specific tumor cell expression but are related to the tumor ecosystem.
Overall, I find this a very interesting concept and have a few suggestions:
Major
1) Although the tool is very flexible the main focus is on immune checkpoints and ligands, however many immune checkpoints are already described and there are ongoing clinical studies or those are already approved (e.g. CTLA4 from the worked example). The authors may demonstrate or discuss that or how novel candidates may be predicted and - as most immune checkpoints are receptor-ligand pairs- how this paired structure may be taken into account as representing a more mechanistic approach.
2) It would be interesting to validate the results using single cell RNAseq data and to find out how ImogiMap can be extended to ligand-receptor pairs that are not restricted to immune checkpoints, as proposed for example by CellPhoneDB.
3) Since users should probably not be limited to bioinformaticians, the authors might consider providing a web application or a better review/testing or introduction to installation, dependencies, etc., as these are the biggest barriers to using the tool.
Minor
Page 3 last line Table S1
Names of the tool should be synchronized (Immogene vs. ImogiMap)

Responses to reviewer comments

COMMSBIO-21-3387A

Mapping the functional interactions at the tumor-immune checkpoint interface

Bozorgui et al.

Responses to Reviewer 1

We thank the reviewer for the constructive comments. We have addressed these points with new analyses using both bulk and single cell RNA expression data and supporting partial correlation analysis of bulk RNA expression data as well as discussion on tumor purity and immune cell types. We have explained our rationale for the current approach with respect to potential alternatives. We also clarified the definition of IAP and how we used IAPs to quantify the interactions between oncogenic and immune processes.

Please note that, to avoid confusion with drug synergy scores, we also replaced the term "synergy score" with "combined action score" for tumor-immune interactions. The score is described both conceptually and mathematically in the manuscript and is identical to the synergy score. Although it is only a name change, we think this is needed as the use of the term "synergy" and its exact description are highly debated.

Reviewer comment 1.1. The algorithm first divides samples based on expression of the inquiry ICP gene and TAP gene, and calculates a synergy score between the two based on the deviation of an IAP to its baseline. Statistically, this can be done much more elegantly through condition correlation, or a simple regression model accounting for both TAP and IAP levels, assuming ICP gene expression level is the response variable.

Author Response 1.1. We agree that other approaches such as partial correlations or regression could also be used to identify interactions between ICP and TAP genes. Indeed, we have implemented other bioinformatics methods that benefit from various algorithms such as LASSO-based feature selection (Li et al, Cancer Discovery, 2022), partial correlations (Wang et al, Biorxiv, in revision, 2021), informatics-based signaling network analysis (Yan et al, Cell Reports, 2022), statistical physics (Korkut et al, 2015, Elife), and machine learning (Yuan et al, 2021, Cell Systems) for inferring biological interactions in different contexts. Each of these methods provide unique advantages such as quantitative predictions of responses to previously untested perturbations in individual samples or building interaction models that are less affected by confounding factors. To address reviewer's comment, we have added the paragraph to the discussion section:

(Discussion section line 323. Also, please see line 369 for a discussion on other immune-oncology algorithms)

"Diverse methods for inference of biological interactions (e.g., signaling interactions, oncogenic co-alterations, and immune relations) have been implemented by our and other groups, a few examples being pairwise or partial correlations, database-driven informatics approaches, regression models, ordinary differential equations, stochastic gradient descent for predictive machine learning models and more recently deep learning approaches (Korkut et al 2015, Li et al 2022, Yuan et al 2021, Margolin et al 2006, Wang et al 2021, Ma, et al 2018). Each of these methods provides unique advantages such as quantitative predictions of responses to previously untested perturbations in individual samples or building interaction models that are less affected by confounding factors. The ImogiMap scheme provides a unique set of advantages. First, ImogiMap can be applied with sparse data as it relies on the calculation of pairwise scores

for immune and tumor processes. This is an important feature as sparse data is common in translational and clinical settings, limiting the implementation of sophisticated machine learning methods. Second, the method does not require rich drug perturbation response or temporal data. Although the use of perturbational and temporal constraints may better enable detection of likely causal interactions, the baseline datasets are still able to capture statistically validated associations. Third, the stratification of patients may enable the selection of relevant patient sub-cohorts based on the co-associations of immune checkpoints and tumor-related events. Such stratification and patient sub-cohort selection may be highly useful in precision oncology applications while partial correlation- or regression-based methods do not immediately lead to a feasible strategy for patient selection. The relatively simple implementation of ImogiMap also enables the incorporation of versatile combined action scores as well as statistical validation schemes. In conclusion, the ImogiMap method is a simple, versatile, and yet informative method for quantitative characterization and statistical validation of the higher-order interactions between oncogenic and immune events."

Reviewer comment 1.2. A more critical issue is data interpretation. In their example (Fig 2D), they show ICP genes are overexpressed, and they suggest CD70 and CD86 have synergistic interaction. However, both genes are highly expressed by APCs, and their "interaction" could simply reflect their co-expression by the same set of immune cells. In fact, all the ICP genes in this example show high expression, suggesting a common covariate, which I suspect is tumor purity.

Author Response 1.2. We agree with the reviewer that common covariates should be investigated when interpreting combined action (previously "synergy") scores. To improve interpretation of the results and address the potential roles of tumor purity and co-expression within the same set of immune cells, we have taken a systematic approach that involves more detailed quantitative analyses of RNA expression in bulk and single cells as well as a more detailed discussion.

1.2.a. Co-expression analysis: To address whether both genes are highly expressed in same immune cell types, we computed the correlation of ICP expressions with immune cell types as well as overall infiltration.

(Results section line 235)

"Next, we asked whether the interaction between CD70 and CD86 is due to a co-expression in or co-association with identical immune cell types. Although both immune regulators are associated with overall immune infiltration (R=0.55 and 0.64 for CD70 and CD86, respectively) as expected, we asked whether they are differentially associated with specific immune cell types. In the absence of comprehensive single cell omics data, we calculated the partial correlations between CD70 and CD86 genes with diverse immune cell type fractions based on RNA expression profiles of tumors from endometrial cancer patients. This is an indirect measure of how each receptor engages with different immune cell types and does not provide definitive proof of their expression sites. We preferred the partial correlation metric as it enables elimination of confounding factors from other random variables while quantifying the direct association between two entities. CD86 is linked with macrophage fractions (both M1 and M2) as well as with regulatory T-cells and activated CD4+ memory T-cells, while CD70 is linked with regulatory T-cells and CD8+ T-cells (Table S7). The partial correlation patterns for the two ICPs suggest differential associations with immune cell types and possible expression patterns in overlapping (likely in regulatory T-cells) but mostly distinct cell types including macrophages, CD4+ memory T-cells, and CD8+ T-cells. The nonuniform associations of CD70 and CD86 expression with different immune cell types suggest the interaction between the two immune stimulators is not an artifact of a uniformly affecting intrinsic tumor impurity manifested as immune infiltration or extrinsic

impurity that may arise from sample collection (See Aran et al, 2016 for a detailed discussion of tumor impurity). This argument is also supported by our statistical validations that demonstrated a robust, specific, and significant interaction between the CD70 and CD86 compared to other immune checkpoints."

1.2.b. Single cell RNA expression. We also added a similar tumor-immune interaction analysis for breast cancer (Figure 3 in the manuscript) and analyzed the expression patterns of the genes involved in statistically validated interactions (e.g., CD70 and CD274) using single cell RNASeq data. The scRNA provides direct information on the co-expression of the interacting tumor and/or immune genes.

(Results section line 278)

"We addressed whether the interaction between CD70 and CD274 may be due to co-expression patterns in identical cell types. We used two sets of single cell RNA sequencing data (Azizi et al. 2018; Gao et al. 2021) to evaluate the mutual exclusivity of gene expressions in the breast microenvironment. We used imputed data from 45,000 immune cells from eight breast carcinoma patients (Azizi et al. 2018), and identified 600 cells that are CD70+ or CD274+ (figure 3E). The single cell RNA sequence analysis suggests that CD70 and CD274 are not co-expressed in identical immune cell types. CD70 is expressed in the majority of CD8+ T-cells and B-cells while CD274 has higher expression in Mast cells, CD4+ T-cells, and macrophages (Figure 3). In addition, CD274 expression is higher in macrophages while CD70 is more abundant in regulatory T-cells (Figure 3E). Both genes are expressed in similar degrees in CD4+ memory T cells. This is consistent with the partial correlation calculations using bulk RNA expression data (source: Breast Cancer TCGA) in which expression of CD274 is linked to the presence of activated and resting CD4+ memory T-cells as well as M1 macrophages, and expression of CD70 is correlated with activated CD4+ T-cells and regulatory T-cells (Table S8). In a separate analysis, we used raw RNA-seq data from around 9000 cells originating from 5 TNBC patients (Gao et al. 2022) and identified around 440 cells that were CD70+ or CD274+ (Figure 3F) and confirmed lack of extensive co-expression in identical cells. Our observations showed that CD70 and CD274 are co-associated with IFNG expression and are predominantly expressed in different immune cell types within the tumor microenvironment."

1: Differential association of CD86 and CD70 with IFNG levels, immune cell types and infiltration in UCEC:

Partial correlations: Table S7. Partial (spearman) correlation values of CD86 and CD70 with immune cell types in UCEC patients. For each gene-immune cell type pairwise partial correlation is calculated in the presence of other immune cell types.

gene	Immune cell types	Partial correlation	Pvalue(FDR adjusted)
CD86	Macrophages.M2	0.272	6.97e-06
CD86	Macrophages.M1	0.215	1.34e-03
CD86	T.Cells.Regulatory.Tregs	0.215	1.36e-03
CD86	T.Cells.CD4.Memory.Activated	0.200	4.51e-03

-

gene	Immune cell types	Partial correlation	Pvalue (FDR-adjusted)
CD70	T.Cells.CD8	0.189	9.76e-03
CD70	T.Cells.Regulatory.Tregs	0.172	3.17e-02

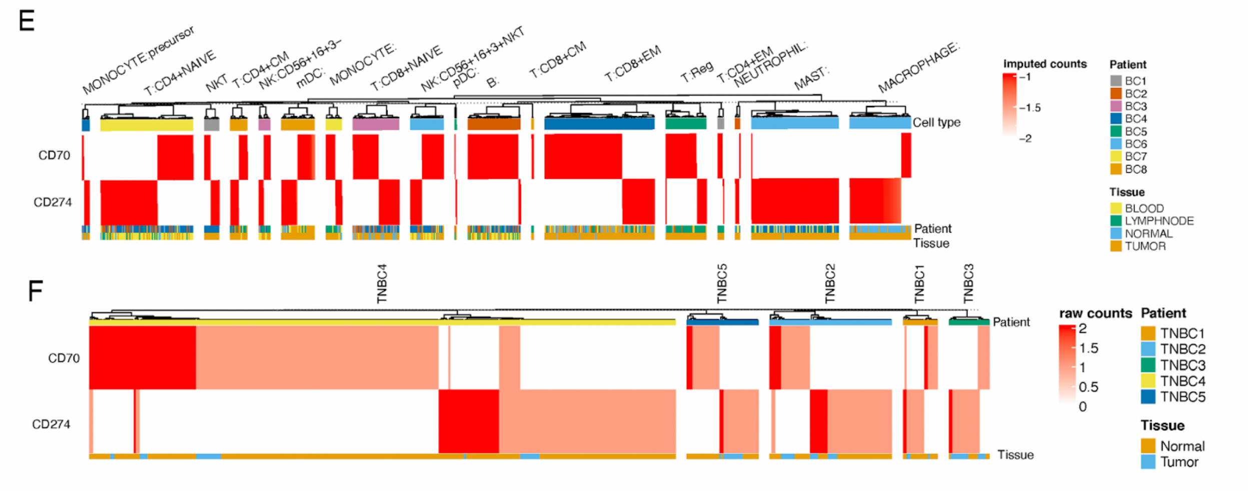

Reviewer comment 1.3. The manuscript did not clearly explain why engaging IAP helps find synergy between ICP and TAP genes. In practice, most of the IAP features are just immune cell abundances predicted by CiberSort. The definition of IAP is obscure. For instance, EMT is considered an IAP. If this is based on the literature, there should be many more pathways/processes that have been associated with tumor immunity.

Author Response 1.3. We agree that a better explanation of the definition of IAP and why we focus on the synergy between ICP and TAP genes will improve the manuscript. We have added the following paragraphs to clarify IAPs and the rationale for engaging IAPs for tumor-immune interactions.

1.3.a. On the definition of IAP:

(Results section line 114)

“We define IAPs as any quantifiable immune-associated event that can potentially modulate tumor immunity and responses to immunotherapy. In our framework, IAP levels serve as a metric to infer the potentially functional ICP-TAP interactions. Here, we have focused on enrichment of immune cell types that may have differential impact on immune regulation as well as Immune cell infiltration (leukocyte fraction) (Hoadley et al. 2018), tumor mutation burden, epithelial mesenchymal transition (EMT) status (Mak et al., 2016), vascularization (Masiero et al., 2013), T cell inflammation signature (Ayers et al., 2017), and IFNg expression (Gao et al., 2016) (Table S2). Indeed, the choice of IAP depends on disease cohort, therapy type, and the oncogenic processes.”

1.3.b. The rationale for focusing on co-associations of ICP and TAP with IAPs:

(Introduction section line 49)

“Infiltration of tumor niches by immune cells usually leads to the enrichment of a large number of coexisting and likely redundant immune checkpoints which obscures the discovery of immune evasion drivers and the selection of immunotherapy targets. Therefore, a naive analysis of immune checkpoint expression and immune cell identities is partially predictive if not totally futile for the identification of therapeutically actionable drivers of immune evasion (O’Malley et al. 2020, Lu et al., 2019).”

(Discussion section line 317)

“The ImogiMap statistical framework aims to extract the functional immune checkpoints that engage with the tumor ecosystems and modulate the tumor and immune characteristics. We have implemented an integrated approach based on the stratification of patient groups, calculation of a combined action score that quantifies the co-associations of ICP and TAP genes followed by comprehensive statistical validation and network representation.”

Reviewer comment 1.4.A minor issue is the exaggerated wording. I am not convinced this tool can “inform on underlying mechanisms that jointly relate TAPs to … ICPs and IAPs,” nor provide “mechanistic links between tumors and the immune system.” I suggest the authors to tone down their wording.

Author Response 1.4. We agree the referee that the mechanistic insight is beyond the scope of this computational tool. We apologize for the misleading phrases. We have toned down and removed references to mechanistic insight. We emphasized the hypothesis generation on potentially functional immunoregulatory events.

<u>Responses to Reviewer 2</u>

We thank the reviewer for the highly constructive feedback and comments that will improve the manuscript. We appreciate the overall positive comments such as “The statistic analysis implemented in the paper is reasonable and convincing.” and “This is a timely study filling an unexplored field. ImogiMap can help basic and translational researchers to discover novel immune-tumor interactions at a scale not accessible easily by experimental methods.” Below, we address the comments and suggestions from the reviewer. We have included receptor-ligand interaction information to our network models using the cellphoneDB database and clarified the observations on the CD70-CD86 interactions. We have also included new analysis using both bulk and single cell RNA expression data as well as a new application in breast cancers.

Please note that, to avoid confusion with drug synergy scores, we also replaced the term “synergy score” with “combined action score” for tumor-immune interactions. The score is described both conceptually and mathematically in the manuscript and is identical to the synergy score. Although it is only a name change, we think this is needed as the use of the term “synergy” and its exact description are highly debated.

Reviewer comment 2.1. From the paper in figure 2G, the high expression of both CD86 and CD70 is associated with better survival in UCEC patients. It is confusing to read "These findings posit the combinatorial targeting of CD86 and CD70 with agonist agents as a new candidate for immunotherapy with likely improved responses in UCEC patients whose tumors carry high CD86 and CD70 expression. ". Does the author actually propose this gene pair as new candidates for immunotherapy for UCEC patients whose tumors carry LOW CD86 and CD70 expression?

Author Response 2.1. We apologize for this confusing phrase in the manuscript. We have revised this section to clarify and prevent any overclaim regarding combination therapy selection. The revised sentence is

(Results section line 259)

"Moreover, we have observed that co-expression of CD70 and CD86 is associated with improved patient survival. The association with both IFNγ responses and patient survival suggests functional immune co-stimulatory roles for the CD86 and CD70 receptors in overlapping contexts within tumors of UCEC patients. Further studies, however, are needed to identify which CD86 and CD70 expression configurations and co-targeting strategies including agonists of both receptors as well as the addition of a third agent (e.g., an immune checkpoint inhibitor) may be tractable in the pre-clinical and clinical settings."

Reviewer comment 2.2. To inform on the underlying mechanisms, it will be a good idea to strengthen the network visualization by incorporating additional network informatics tools (e.g., protein-protein interaction, ligand-receptor interaction) into your network models as an option for users to visualize/analyze the network.

Author Response 2.2. We thank our reviewer for this insightful suggestion. In line with this suggestion, we have included a parameter for users to add ligand-receptors genes to their initial list of ICP/TAP genes. If checked, ImogiMap searches CellphoneDB database for existing ligand-receptor pairs and adds the corresponding genes to the initial gene lists. We also incorporated ligand-receptor interactions in our network diagram. An example of a network diagram is provided below, where a solid black line is added if two neighboring vertices are found as pairs in cellphoneDB receptor-ligand database. Moreover, we repeated our analysis by including ligand-receptor pairs in our initial gene sets both in UCEC and base-like breast cancer case studies.

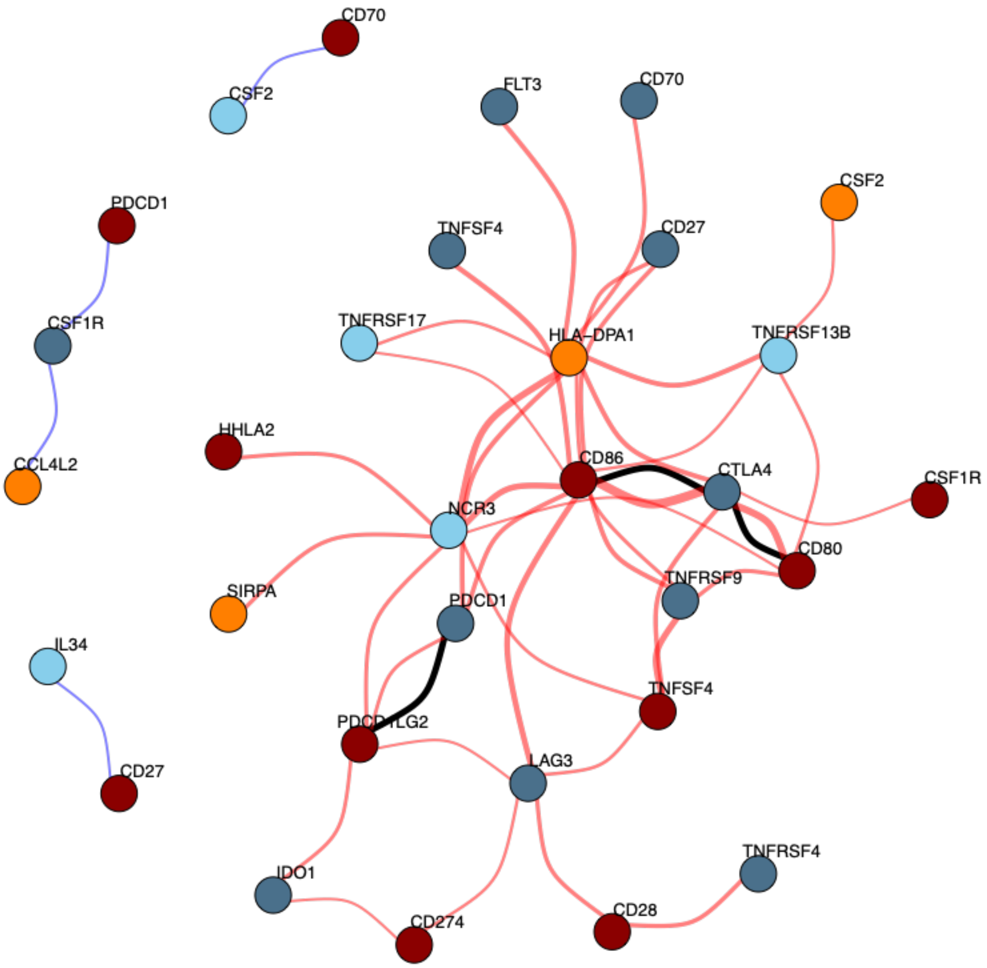

Figure S1. *A Sample graphical network depicting receptor ligand pairs from CellphoneDB as black edges. Red/Blue edges depict combined action scores of immune checkpoints and their corresponding ligand/receptors on fraction of M2 macrophages (IAP) in TCGA LUAD samples (576 samples). The receptor ligand pairs which are also inferred as adjacent vertices, namely PDCD1 and PDCD1L2, CTLA4 and CD80, CTLA4 and CD86 are represented with black edges.*

Responses to Reviewer #3

We thank the reviewer for the insightful review, important suggestions and positive comments such as “The method is statistical sound and well explained” and “Overall, I find this a very interesting concept”. Here, we addressed all referee comments with improvements in the source code, implementation of a web interface and incorporation of receptor-ligand interactions using the CelllPhoneDB database. We included a discussion on how new interactions can be

predicted. We included a single cell RNAseq analysis with focus on the expression patterns of the interacting partners (CD70, CD86, CD274).

Please note that, to avoid confusion with drug synergy scores, we also replaced the term "synergy score" with "combined action score" for tumor-immune interactions. The score is described both conceptually and mathematically in the manuscript and is identical to the synergy score. Although it is only a name change, we think this is needed as the use of the term "synergy" and its exact description are highly debated.

Reviewer comment 3.1. Although the tool is very flexible the main focus is on immune checkpoints and ligands, however many immune checkpoints are already described and there are ongoing clinical studies or those are already approved (e.g. CTLA4 from the worked example). The authors may demonstrate or discuss that or how novel candidates may be predicted and - as most immune checkpoints are receptor-ligand pairs- how this paired structure may be taken into account as representing a more mechanistic approach.

Response 3.1. We agree with our reviewer that including receptor-ligand pairs may provide insight to mechanisms. receptor ligand pairs are incorporated from CellPhoneDB so users can search for such pairs in depth using our tool with information from CellPhoneDB. We have now incorporated known receptor ligand pairs into our calculations and visualization. This provides a guide for selection of events that involve more likely correlated and functional features. Please see the more detailed responses 3.2 and 2.2 how we addressed receptor-ligand interactions. To address how ImogiMap can be used for generation of new hypotheses we added the following sentence to the manuscript:

(Discussion section line 347)

"The flexibility and statistical rigor of the ImogiMap algorithm may enable generation of hypotheses for the discovery of previously unreported physical or functional interactions. Our current implementation is optimized for immune interactions partly due to the immediate need in the field. ImogiMap, however, is not necessarily limited to tumor-immune interactions, and with its flexibility, it enables different modes of discovery. First, the algorithm can be applied to identify diverse mediators that may together impact a given phenotype (predict interacting genes that associate with a particular phenotype). Execution of ImogiMap with a large gene set against a phenotype of interest may generate a list of interactions that are significantly co-associated with the target phenotype. Second, the algorithm can be used to discover novel interaction partners of a gene, whose impact on a given phenotype is already known (discovery of additional regulators of a phenotype with already known mediators). In this case, the ImogiMap can be executed for a pre-defined gene against a large set of candidate genes and the outcomes may guide selection of interactions that mediate a particular phenotype. A major challenge in ImogiMap is selection of likely functional events based on co-associations, which do not dictate causation. This is particularly challenging when baseline, correlative data is used from large patient cohorts as in the case of TCGA datasets. A potential solution to select likely causal interactions is to use time series and perturbation response data. The sequence of expression vs. phenotypic events in time or differential response to perturbations may provide the constraints to establish causality and directionality of interactions. Indeed, the incorporation of such data into ImogiMap is straightforward with minimal design differences. Experimental validation of the predictions, however, is essential to claim a novel discovery regardless of the underlying data modality as well as time and perturbation schemes."

Reviewer comment 3.2. It would be interesting to validate the results using single cell RNAseq data and to find out how ImogiMap can be extended to ligand-receptor pairs that are not restricted to immune checkpoints, as proposed for example by CellPhoneDB.

Author response 3.2. In line with the referee suggestions, we have included a new ImogiMap analysis using breast cancer mRNA data and subsequent single cell RNA expression analysis. The results are given in Figure 3. In summary, we benefited from single cell RNAseq data to address whether the association between CD274 and CD70 emerges due to co-expression in the same cell types. We provided direct evidence that the two ICPs are expressed predominantly in different cell types. Although the two ICPs were expressed in cell CD4+ Naive T-cells, the CD70 was highly expressed in CD8+ T-cells, while CD274 was enriched in mast and macrophage cells.

Although single cell RNA expression data is highly valuable, detailed statistical analyses as in ImogiMap is challenging with scRNAseq data due to small sample sizes. Therefore, we believe analysis of bulk RNAseq data from large patient cohorts carry high value. In the future, we expect to incorporate scRNA to ImogiMap as more data become available.

We agree extension into receptor-ligand pairs is an exciting direction and we have implemented this feature as needed. We have included a parameter for users to add ligand-receptors genes to their initial list of ICP/TAP genes. If checked, imogiMap searches CellphoneDB receptor-ligand database for existing ligand-receptor pairs and adds the corresponding genes to the initial gene lists. We also incorporated ligand-receptor interactions in our network diagram. An example of a network diagram is provided in response 2.2, where a solid black line is added if two neighboring vertices are found in the cellphoneDB receptor-ligand database. Moreover, we repeated our analysis by including ligand-receptor pairs from CellphoneDB in our initial gene sets both in UCEC and base-like breast cancer case studies. This is included into figure 2-3 and explained in the results section.

Although the method can be immediately adapted to any application with custom selected gene sets, we have limited our analyses and scope to a specific domain (i.e., immune checkpoints) for the following reasons. (i) Detection of ICPs that may serve as immunotherapy targets is highly needed and yet challenging due to the redundancy of ICPs in the tumor ecosystems. (ii) Availability of data from sufficiently large patient cohorts that enable detection of ICP-TAP interactions (iii) Increased number of false positives and challenges for controlling FDR rates that would require data from much larger patient cohorts when a large number of receptor-ligand pairs are included.

Reviewer comment 3.3 Since users should probably not be limited to bioinformaticians, the authors might consider providing a web application or a better review/testing or introduction to installation, dependencies, etc., as these are the biggest barriers to using the tool.

Author Response 3.3. We agree that a web interface is needed for at least better presentation of the results as well as the implementation of the computational tool. We have implemented the R-shiny based web interface at

https://bioinformatics.mdanderson.org/apps/imogimap

We have improved the code for usability and with a tutorial. The ready to use code is available at

https://github.com/korkutlab/imogimap.

The web application is user friendly. However, for comprehensive statistical evaluations which need computational power and time, we recommend downloading the source code.

Minor

Page 3 last line Table S1

Names of the tool should be synchronized (Immogene vs. ImogiMap)

Author response. We thank the referee for pointing out this important issue. We have corrected.

REVIEWERS' COMMENTS:

Reviewer #1 (Remarks to the Author):

I appreciate the authors' efforts to add a web interface, single cell analysis, and breast cancer analysis. In addition, the paper benefits from partial correlation that clarifies the origin of co-expression by some of the identified interactions.

A few minor suggestions. First, IAP in the text is noted as "immune associated process", while in Fig 1A it stands for "immune associated phenotypes." It should be made consistent throughout the paper.
Second, in breast cancer data analysis, they show CD70 and CD274 interact, and the two genes are expressed by distinct sets of cells (Fig 3E, F). I think this pattern is clear. However, they limited their analysis to only CD74 or CD274 expressing cells. This could be inadequate because this limitation ignores the total amount of cells, which can affect data interpretation. For instance, Fig 3E shows CD274 is preferentially expressed by MAST cells compared with CD4 naïve T cells. However, if the number of MAST cells identified in the dataset is much higher, the average expression of CD274 may not be interpreted as higher in MAST than in CD4 naïve T cells.

Reviewer #2 (Remarks to the Author):

The manuscript has been greatly improved. The authors have addressed all of my concerns.

Reviewer #3 (Remarks to the Author):

The authors have adequately addressed raised issues:
1. They have added an R Shiny app as easy to use entry point (and also refer to the R code from github as building of bootstrap distributions takes some time), both are working as described
2. They have added receptor-ligand information from CellPhoneDb
3. Included single cell RNAseq data and at least for one example
demonstrated mutual exclusivity of expression in different cell types of the microenvironment and compared to partial correlation analyses
4. The manuscript has been significantly improved. Although the logic of the explanations/definitions given are comprehensible, it is still not very intuitive that, for example, EMT is referred to as an immune-associated phenotype and T-cell dysfunction as a tumor-associated process.
